# Leveraging Curation Among *Escherichia coli* Pathway/Genome Databases Using Ortholog-Based Annotation Propagation

**DOI:** 10.3389/fmicb.2021.614355

**Published:** 2021-03-08

**Authors:** Suzanne Paley, Ingrid M. Keseler, Markus Krummenacker, Peter D. Karp

**Affiliations:** Bioinformatics Research Group, SRI International, Menlo Park, CA, United States

**Keywords:** genome annotation, *Escherichia coli*, orthologs, database curation, annotation propagation

## Abstract

Updating genome databases to reflect newly published molecular findings for an organism was hard enough when only a single strain of a given organism had been sequenced. With multiple sequenced strains now available for many organisms, the challenge has grown significantly because of the still-limited resources available for the manual curation that corrects errors and captures new knowledge. We have developed a method to automatically propagate multiple types of curated knowledge from genes and proteins in one genome database to their orthologs in uncurated databases for related strains, imposing several quality-control filters to reduce the chances of introducing errors. We have applied this method to propagate information from the highly curated EcoCyc database for *Escherichia coli* K–12 to databases for 480 other *Escherichia coli* strains in the BioCyc database collection. The increase in value and utility of the target databases after propagation is considerable. Target databases received updates for an average of 2,535 proteins each. In addition to widespread addition and regularization of gene and protein names, 97% of the target databases were improved by the addition of at least 200 new protein complexes, at least 800 new or updated reaction assignments, and at least 2,400 sets of GO annotations.

## 1. Introduction

Manual curation of biological databases is a time-consuming and moderately expensive (Karp, 2016b) task, requiring biological expertise, attention to detail, and the ability to sift through and evaluate the experimental literature. However, the outcome of all this applied effort and expertise is that expert manual curation remains the gold standard of database quality (Keseler et al., 2014). Cheaper automated text-mining systems, while suitable for certain limited applications, are not yet capable of making the determinations required to populate rich, complex, multi-datatype databases such as those in the BioCyc collection (Karp, 2016a). What automated tools do very well, however, is make inferences based on patterns, enabling knowledge gained in one sphere to be extended to other related situations. Here we describe an automated method that propagates curated information from one Pathway/Genome Database (PGDB) to other PGDBs within BioCyc. We have applied the method to propagate curation from the EcoCyc database to databases for 480 other *E. coli* strains in BioCyc, thereby leveraging limited curation resources to greatly increase the value of BioCyc.

EcoCyc (Keseler et al., 2017) is a comprehensive PGDB describing the genes, metabolism, and other functions of *E. coli* K-12 MG1655, the best-studied bacterial model organism. EcoCyc is the product of nearly three decades of manual curation, with 39,000 citations to the primary literature, and is thus the gold standard for PGDBs in terms of quality, accuracy, and depth and breadth of coverage.

BioCyc (Karp et al., 2017) is a collection of PGDBs for over 18,000 organisms. With the exception of EcoCyc, all the other single-organism PGDBs in the collection were generated from their annotated genome via the PathoLogic software (Karp et al., 2015), which creates a PGDB, imports the genome and proteome of the organism, assigns enzymes and transporters to reactions, and infers metabolic pathways. A handful of these PGDBs have undergone varying amounts of additional manual curation, but the vast majority have received no curation, and thus their quality depends entirely on the quality of the original annotation and our software's ability to derive useful information from that annotation. Four hundred and eighty of these databases are for other *Escherichia coli* strains.

For multiple reasons, an automatically generated PGDB might contain errors or omissions. The sequence-annotation pipelines that produce the input to our PathoLogic software vary in their degrees of coverage, sophistication, and accuracy. Some pipelines include gene names, whereas others do not. Some include EC numbers and/or GO annotations, which others omit. Sequence annotation, which is based on a variety of methods (including sequence similarity, protein family assignment and protein domain identification, Haft et al., 2017), is vulnerable to transitive annotation errors and errors in specificity (Promponas et al., 2015), and may not reflect the most up-to-date knowledge. Our PathoLogic pipeline also has several possible sources of error, including sensitivity to variations in protein function descriptions, and a lack of specificity in some cases when assigning reactions to proteins. In a curated PGDB such as EcoCyc, curators correct such errors, as well as update the data to reflect new or changed knowledge, such as a changed gene name or a newly discovered protein function. Thus, we hypothesized that data propagation of data from a highly curated PGDB to an uncurated or less well-curated PGDB for a closely related organism (i.e., another strain of the same species) would result in a significant improvement in quality and coverage, at minimal cost. We have developed such a propagation method and applied it to propagate curation from EcoCyc to the 480 other *E. coli* PGDBs.

One might argue that any software that uses sequence similarity and homology-based classifiers to annotate genomes is performing a sort of ortholog-based propagation of annotations. Our approach differs from genome-annotation pipelines in several respects. First, our approach is focused on *updating* existing annotations in the target genome, based on annotations in the source genome with experimental providence. Second, our approach propagates only for proteins that are orthologs, as opposed to other kinds of sequence similarity. Third, our approach propagates a wider array of information than genome-annotation pipelines: gene name, product name, GO terms, reaction assignments, and membership in protein complexes. Fourth, our approach propagates only from a well-curated gold-standard strain to other strains of the same species, whereas annotation pipelines propagate across longer evolutionary distances.

Some other approaches that propagate curated information across multiple organisms include PAINT and HAMAP. PAINT (Gaudet et al., 2011) is a software tool for propagating GO annotations based on phylogenetic trees. For each gene family, a phylogenetic tree is constructed and curated with function gain and loss events. Once such a tree is generated and curated for a gene family, new sequences can be inserted using the TreeGrafter (Tang et al., 2019) tool, and annotations can be readily inferred (and updated as curation is updated) for any sequence in the family. HAMAP (Pedruzzi et al., 2015) combines manually curated protein family profiles with a set of curated annotation rules to assign and update a wide range of information fields similar in breadth to our own approach, including gene and protein names, function, catalytic activity, cofactors, subcellular location, protein-protein interactions, protein features and GO terms. This tool is used as part of the UniRule (MacDougall et al., 2020) system to propagate annotations to unreviewed proteins in UniProtKB. The advantage of these approaches is that they support annotation propagation across a wide range of organisms. However, the level of curation required to support these methods must also encompass a similarly wide range of organisms in order to properly capture the boundaries of association between individual functions and sequences. Creating the HMM models used by HAMAP is also time-consuming and requires some minimum number of sequences. In contrast, by applying our approach only within the strains of a single species, and only to identified orthologs that pass a strict set of filters, we can propagate information with a high degree of confidence despite having only a single curated gold-standard strain.

## 2. Materials and Methods

Most but not all of the genomes that make up BioCyc were originally annotated by and downloaded from RefSeq (Haft et al., 2017). Before starting this project, we rebuilt all *E. coli* PGDBs in BioCyc from the newest RefSeq annotations if newer annotations were available than those already present in BioCyc, to ensure that our analyses of the changes made by the propagation process reflected improvements over the most recent RefSeq annotations.

Our data propagation method relies on mapping each protein-coding gene in the target PGDB (to which information will be propagated) to its corresponding ortholog in the source PGDB (in this case EcoCyc). Assuming a unique ortholog can be identified, we then apply a set of quality filters to the pair to determine whether or not data for this gene and its product should be propagated. Only those pairs that pass all filters will have their data propagated. These filters are designed to be conservative, in that we prefer not to propagate information at all for some gene rather than to risk overwriting correct information with incorrect information.

The BioCyc project computes orthologs between two genomes using bidirectional BLAST comparisons across their proteomes. Two proteins are inferred to be orthologs if they are best bi-directional BLAST hits with both *E*-values less than 0.001. The “best” hit(s) of protein A in proteome *P*_*B*_ is defined by finding the minimal *E*-value among all hits in proteome *P*_*B*_ in the BLAST output. There could be hits to multiple proteins in proteome *P*_*B*_ that share that same minimal *E*-value. In other words, ties are possible, as in the case of exact gene duplications. All ties are included in the final set of orthologs used by BioCyc. Thus, protein A could have multiple orthologs in *P*_*B*_, such as if multiple proteins *B*_1_, *B*_2_, etc., exist in *P*_*B*_, and have exactly the same regions aligned against protein A. BioCyc does not calculate paralogs.

When a protein in the target PGDB maps to multiple orthologs in the source PGDB, the propagation algorithm attempts to determine the best candidate based on synteny (preferred) or gene names. Synteny has been identified as an important factor for distinguishing among multiple ortholog candidates (Fouts et al., 2012). We consider a pair of ortholog gene products *S* (gene in the source PGDB) and *T* (gene in the target PGDB) to pass the synteny test if the product of either of the two genes directly adjacent to *S* is an ortholog to the product of either of the two genes directly adjacent to *T*. If none of the multiple orthologs pass the synteny test, we check to see if any of them have the same gene name as does *S*. Gene names are a less reliable indicator than synteny, because many genome annotations omit them and they depend on accurate annotation, but if they are available, we use them. If we cannot determine a single best ortholog match *S* for a gene *T*, then *T* is rejected as a possible target for data propagation.

Once a candidate ortholog pair *S* and *T* has been identified, it must pass all of the following filters in order to qualify for propagation.

- ***T* lacks an experimental or literature-based evidence code**. If the target PGDB has undergone any curation, we do not want to risk overwriting that work. Any curated changes to *T* or its associated objects should be accompanied by a corresponding evidence code, so any such evidence code will prevent propagation. The vast majority of our PGDBs have not undergone any manual curation so this filter is rarely triggered.
- ***S* has an experimental or literature-based evidence code**. To avoid transitive annotation errors, data will only be propagated from genes that have some experimental or literature-based evidence code.
- ***P*-value test**. The sequence similarity *P*-value, generated during the ortholog computation, must not exceed 10^−10^ (this is a configurable threshold). The ortholog computation calculates the *P*-value as the arithmetic average between the two *e*-values, which resulted from the best bi-directional BLAST hits. Matches are better, the smaller the values are.
- **Length test**. The two sequences must not differ in length by more than 10% (this is also a configurable threshold).
- **Complex component test**. If *S* is a component of a heteromultimeric complex, then the software must identify orthologs for all other components of that complex in the target PGDB in order to pass this test.

Not all attributes of a protein are appropriate for propagation. For example, the curated textual summaries can be strain-specific and so should not be propagated. Other factors such as enzyme kinetic properties and regulation are also likely to be strain-specific. Thus, even once we have successfully identified an ortholog pair for propagation, we must be selective about which attributes get copied. We chose to confine propagation to the following attributes: gene name and synonyms, product function name and synonyms, GO term assignments, reaction assignments, and membership in a heteromultimeric complex and the reaction assignments of the complex. Only those GO terms with experimental evidence codes in EcoCyc are propagated unless the protein sequences of the two orthologs are identical, in which case all GO terms are propagated. All propagated GO terms are given ISO evidence codes with references to the source ortholog. When we refer to propagation of a gene or protein in this paper, we are referring to the propagation of all of the above-listed data attributes for the gene and its protein product. When propagation occurs, a history note is generated for the target gene that identifies the source gene, and lists all the fields propagated and their prior values. This history note is visible on the web page for the target gene. A particular attribute is not considered to have been propagated unless the new value is actually different from the old value.

Considering all the above criteria, we iterate through each of the protein-coding genes in each target PGDB and, if an appropriate source ortholog is identified that passes all the filters, propagate the designated fields. Once propagation is complete, we re-run the PathoLogic pathway inference algorithm to ensure that the set of pathways predicted for the organism is consistent with the updated reaction assignments. We also generate a report (see Supplementary Material) that (a) provides summary statistics, (b) lists which fields were propagated for every protein whose data was propagated, and (c) lists the reasons why not for every protein for which no data was propagated.

After propagation, we asked multiple biologists to review a small number of target PGDBs after propagation to verify that information was being propagated appropriately and that no obvious errors were introduced.

## 3. Results

**Figure 1:**
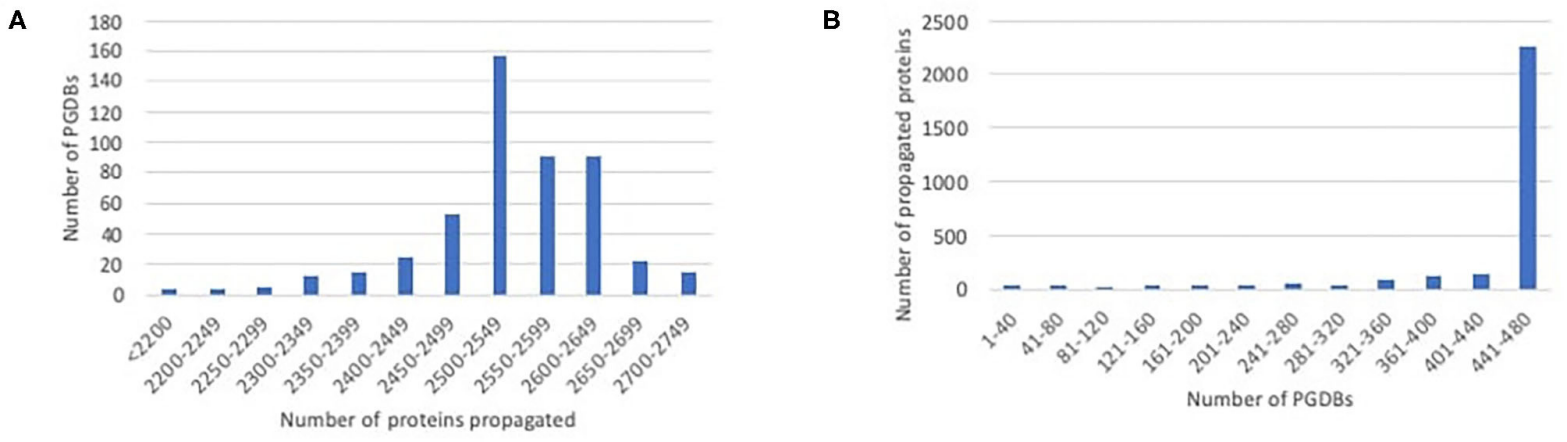
Histograms showing the distribution of propagated proteins. **(A)** The number of proteins propagated per PGDB. For example, the largest number of PGDBs each had 2,500–2,549 proteins propagated to them. **(B)** The number of PGDBs each protein was propagated to. Most proteins that were propagated in any case were propagated to nearly all PGDBs, with smaller numbers propagated to only a subset of PGDBs.

We ran our propagation tool to propagate data from EcoCyc to each of the 480 PGDBs for other *E. coli* strains. EcoCyc currently has 2,848 protein-coding genes with known sequence and associated experimental or literature-based evidence codes, so this number constitutes an upper limit on the number of proteins that can potentially be propagated to any PGDB. The number of proteins that were propagated to any particular PGDB ranged from 1,935 to 2,743, with the majority of PGDBs having 2,450–2,650 propagated proteins, as shown in Figure 1A. Most proteins that were propagated in any case were propagated to nearly all of the target PGDBs, as shown in Figure 1B. A smaller number of proteins were propagated to only a handful of PGDBs. Some in the latter category are proteins encoded by prophages (e.g., *argF, appY*) that presumably lack orthologs in many strains. This category also includes some smaller genes whose small length differences are more likely to cause an ortholog to fail the length filter (e.g., *ldrA, ivbL*), and some members of large complexes that lack an ortholog for one or more complex member (e.g., *gspC–O*), causing all members to fail the complex filter.

**Figure 2:**
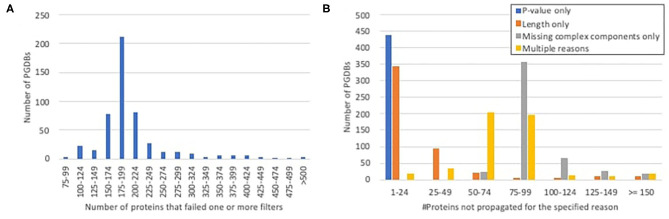
Histograms showing the number of proteins per PGDB for which unique experimentally characterized orthologs were identified in EcoCyc, but which were not propagated because they failed to pass the other filters. **(A)** The total numbers of proteins per PGDB that failed any filter. **(B)** The numbers of proteins per PGDB that failed either filter alone or multiple filters.

In most PGDBs, of the gene products for which unique, experimentally characterized orthologs were identified, roughly 150–250 were not propagated, because they failed to pass one or more of the other required filters (Figure 2A). When we looked at which filters were failed (Figure 2B), very few proteins failed solely based on *P*-value. A handful of proteins failed solely based on length differences. Approximately a third to a half of the proteins failed multiple filters. The rest were components of heteromultimeric complexes that lacked identifiable orthologs for one or more other subunit. This includes all ribosomal components, which were not propagated to any PGDBs, since our ortholog computation excludes RNA. We considered the possibility of working around this filter for RNA-containing complexes, but because such a workaround would raise potentially significant complications, and because ribosomal proteins generally are already well-annotated, we decided that the potential benefit was not worth the effort.

## 4. Discussion

**Figure 3:**
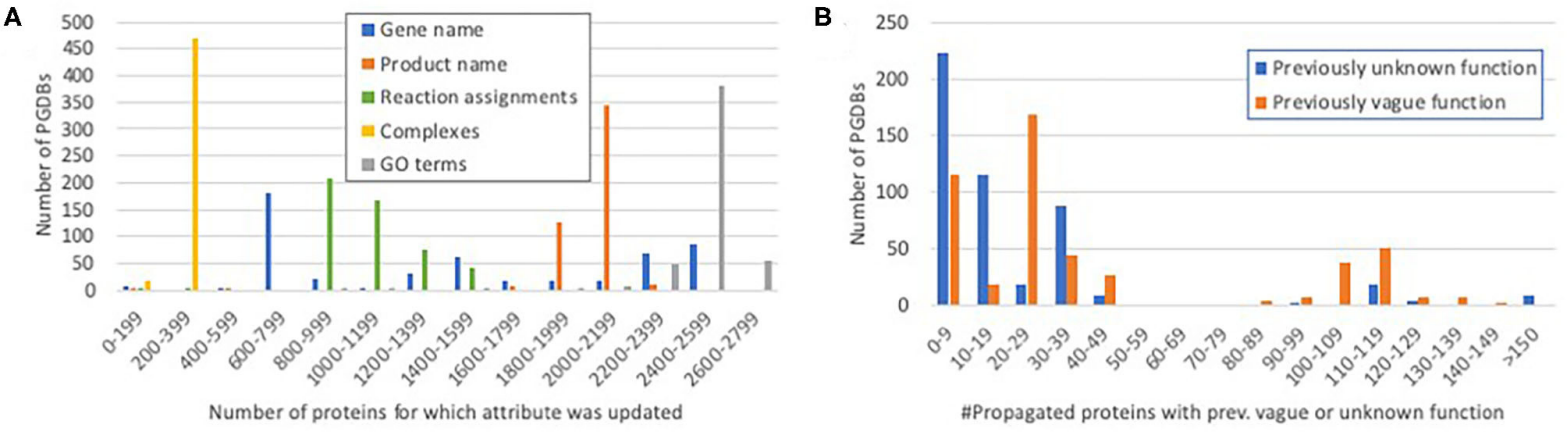
Histograms showing the distribution of propagation of significant protein attributes. **(A)** The number of proteins for which each potentially significant attribute was propagated per PGDB. For example, most PGDBs had 200–399 complexes propagated and 1,800–2,199 protein names propagated. **(B)** The number of propagated proteins per PGDB that had previously vague or unknown function (see Discussion) and acquired a more specific function during propagation.

For the genes and proteins whose annotations were propagated, we were interested in distinguishing between substantial changes—those that improve a user's understanding of the gene function—and relatively minor changes, such as addition of a synonym or small wording change. Figure 3A shows the distribution of propagation of fields that can represent meaningful changes. Nearly all propagated proteins were assigned new GO annotations. Most PGDBs had 200–250 complexes propagated—close to the number of curated heteromultimeric complexes in EcoCyc (298).

Most PGDBs received updated reaction assignments for 900–1,100 proteins. This set includes both cases in which new or different reaction assignments were added to a gene product and cases in which spurious reaction assignments were removed. For example, *ybiV* is a sugar phosphatase shown to act on a range of substrates. In EcoCyc, it is assigned to a generic sugar phosphatase reaction. However, because the protein belongs to a larger family of haloacid dehalogenase hydrolases, in many PGDBs it was previously assigned to a set of dehalogenase reactions. These reactions were replaced with the EcoCyc reaction. In some other PGDBs where the gene had been correctly annotated as a sugar phosphatase, that annotation was not considered sufficiently specific for PathoLogic to assign to any reaction, so the reaction from EcoCyc was added during propagation. Many enzymes catalyze secondary reactions in addition to their main activity. For example, *adh*, adenylate kinase, has demonstrated the ability to fulfill the role of a general nucleoside diphosphate kinase, and therefore in EcoCyc has been assigned seven other reactions in addition to its main adenylate kinase activity. In most other PGDBs, this gene product was previously assigned only to its main activity, and the secondary reactions were added during propagation. Spurious reaction assignments can result from errors or imprecision in either the genome annotation or in the PathoLogic reaction assignment algorithm. For example, *phoU*, a regulator of the pho operon and not an enzyme at all, had in a number of strains been annotated as a transport accessory protein and had therefore been assigned a phosphate transport reaction. This reaction was removed upon propagation.

With respect to gene names, most PGDBs fall into one of two subsets: those in which 700–800 gene names were propagated, and those in which 2,300–2,500 gene names were propagated. Many annotated genomes lack gene names altogether (they were annotated by the NCBI prokaryotic annotation pipeline) and these genomes constitute the latter set. The genes from the former set whose names were propagated include some genes that, for whatever reason, were not assigned a name even in genomes where other gene names were assigned, as well as genes with assigned names where the names differ from those in EcoCyc. For example, in most other *E. coli* PGDBs, the orthologs of the EcoCyc gene *cysG* were annotated as *cobA* (there is no gene named *cobA* in EcoCyc—*cobA* is a synonym for the gene *btuR*). The EcoCyc gene formerly named *yehT* had its named changed to *btsR* several years ago (*yehT* remains a synonym), but most of the other *E. coli* genome annotations still use the name *yehT*.

Most PGDBs had updates to more than 1,900 protein names. Because such updates can include both minor changes in wording that do not affect meaning and more significant changes, programmatically distinguishing between these cases is not easy. However, we wrote a program that categorizes certain product name patterns as corresponding to unknown function (e.g., “hypothetical protein,” “orf”), and certain other patterns as corresponding to a vague, non-specific function (e.g., “transporter,” “oxidoreductase,” and “regulatory protein”). We counted the number of genes that had unknown or vague function assignments before propagation that were converted to more specific and informative function assignments by propagation. A histogram of the results is shown in Figure 3B. Most PGDBs had fairly small numbers of proteins with newly acquired specific function assignments, but a quarter of the PGDBs had more than 100 such cases, demonstrating that the propagation adds significant new functional information beyond the latest available RefSeq annotations. One example is *yqfB*, N^4^-acetylcytidine aminohydrolase, which was annotated with unknown function in 30 PGDBs, and as ASCH domain-containing protein in most others. The updated functional description is a significant upgrade in both cases. Another example is *nanT*, N-acetylneuraminate:H^+^ symporter, which in nearly 200 PGDBs is annotated merely as MFS transporter (considered vague), providing no information about the transported substrate, and in most other PGDBs as sialic acid transporter, which specifies the substrate but not the mechanism (H^+^ symport).

**Figure 4:**
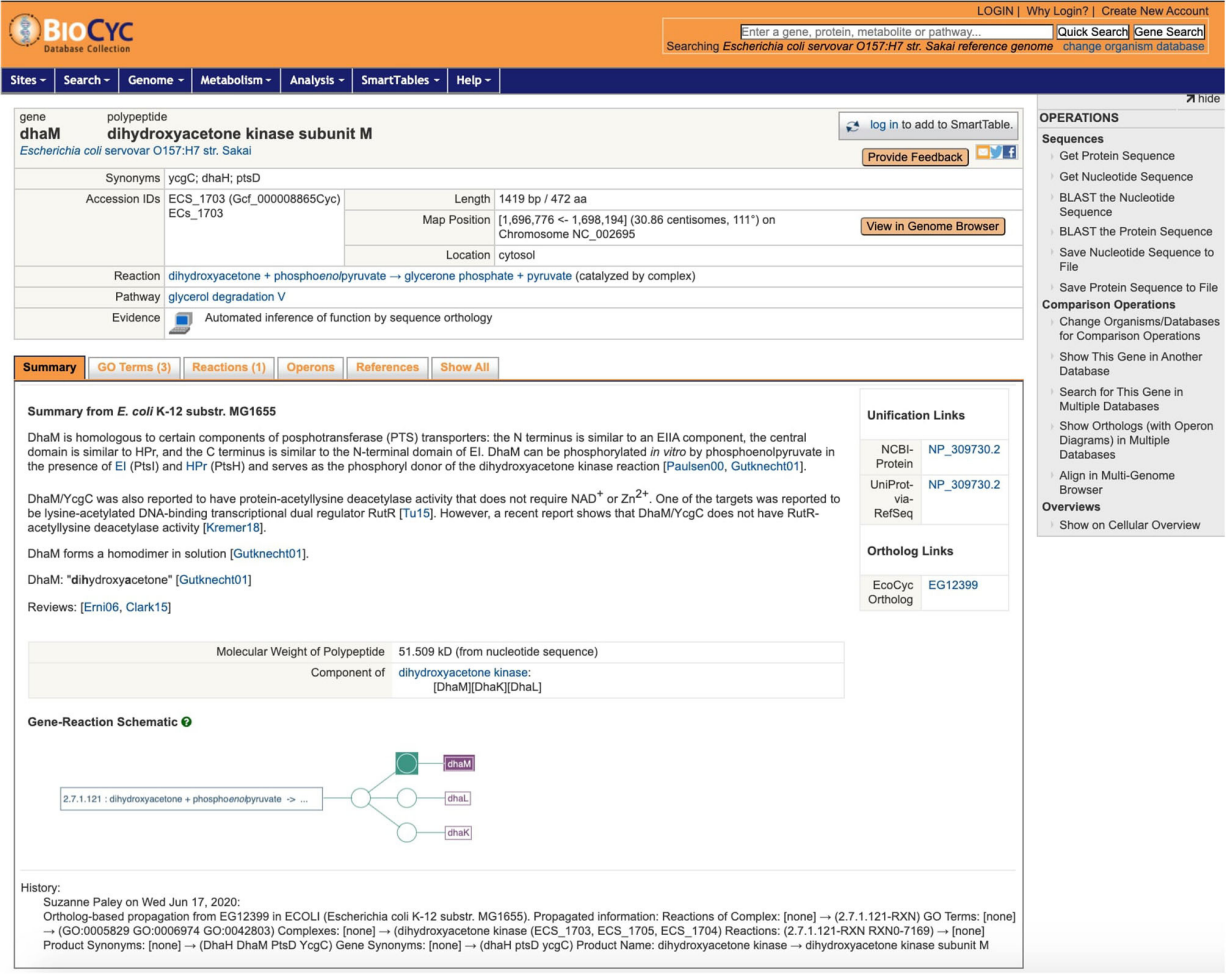
A sample gene page, for *dhaM* in *Escherichia coli* serovar O157:H7 Sakai, after propagation from EcoCyc. Note the textual summary with literature references from K–12 MG1655, the link to the gene's ortholog in EcoCyc, and the history note that briefly describes the changes.

One of the strengths of EcoCyc is its detailed textual summary for each curated gene, a mini-review carefully culled from the primary literature, with citations to the primary sources. As already stated, because this information can be strain-specific, the textual summaries are not copied as part of ortholog propagation. Nonetheless, this information can provide valuable context, and by adding it to the gene pages for other strains, we dramatically improve the value of those gene pages to the user. Thus, when we propagate a gene product to its ortholog in another PGDB, we record that fact in the target gene. A new feature added to the gene page for the target gene is that, in addition to its own textual summary (usually extracted from the genome annotation), we now also include the summary from the source gene, clearly marked with the strain it came from, as shown in Figure 4. This addition enables a user to browse the strain they are interested in without constantly having to refer back to EcoCyc.

After ortholog propagation was completed, we re-ran our pathway-prediction algorithm (Karp et al., 2011) on the updated PGDBs. We do not assume that the presence/absence of a pathway in EcoCyc determines its presence/absence in other *E. coli* PGDBs. Rather, the determination of whether or not a pathway is inferred in a PGDB depends on the catalyzed reactions in that PGDB. Given the many changes to the reaction complement of each PGDB, however, we expected significant changes in the set of inferred pathways, and this is in fact what we observed. Many new pathways were inferred, only some of which are present in EcoCyc. For example, the L-lyxose degradation pathway is a four-reaction pathway in EcoCyc whose key reaction is the first reaction, the conversion of L-lyxopyranose to L-xylulose. In EcoCyc, this reaction is a secondary activity of *rhaA*, L-rhamnose isomerase. Propagating this activity to the corresponding orthologs in other PGDBs enables this pathway to be predicted in those PGDBs. Microcin B17 biosynthesis is a three-reaction *E. coli* pathway that is not present in EcoCyc because the genes for two of the steps normally reside on a plasmid that is not present in MG1655. The remaining reaction is catalyzed by the complex TldDE (both genes were previously usually annotated as just metalloproteases, without indication of their specific function), and this reaction was propagated to other PGDBs, enabling this pathway to be predicted in most PGDBs. Unfortunately, in some cases this is likely an erroneous prediction, since the presence of this one reaction, which is unique to this pathway, causes the pathway to be predicted even in strains that lack the plasmid. The propensity of the PathoLogic pathway predictor to predict pathways even if not all enzymes have been identified is intentional, to accommodate the typical incompleteness of genome annotations, but it does sometimes lead to over-predictions.

In other cases, pathways that were previously erroneously inferred were removed. For example, in some organisms, but not so far demonstrated in *E. coli*, a secondary activity of the enzyme sulfate adenylyltransferase is the adenylylation of selenate. For this reason, PathoLogic erroneously assigns this secondary reaction to the sulfate adenylyltransferase subunits CysN and CysD, leading to the prediction of a selenate reduction pathway. Removing the selenate reaction from the CysDN complex means that the selenate reduction pathway is appropriately no longer predicted.

In conclusion, we have developed a method for propagating knowledge from a well-curated PGDB to computationally generated PGDBs for many other related strains. We are confident that the quality control filters we have put in place minimize the likelihood that this process will introduce new errors into the target PGDBs. We have shown that the increase in value and utility of the target PGDBs after propagation is considerable, with most PGDBs receiving more than 200 new protein complexes, more than 800 new or updated reaction assignments, more than 2,400 sets of GO annotations, and widespread addition and regularization of gene and protein names. This software enables us to leverage the limited curation resources of the EcoCyc project, extending their applicability more widely to a large number of strains.

We have considered applying this propagation approach more broadly, to propagate curated knowledge from EcoCyc to other related genera such as *Salmonella* or *Shigella*. However, the greater the phylogenetic distance between source and target PGDB, the greater the likelihood for introducing error. For example, gene-name conventions vary from species to species, so propagation of that field may be problematic. Nonetheless, we may explore variations of our method in the future to support this extended use case. We are already using this method on a limited basis to propagate curated information from some BioCyc Tier 2 PGDBs (those that have undergone some curation, but substantially less than EcoCyc, meaning many fewer genes will be eligible for propagation) to related strains.

## Data Availability Statement

The EcoCyc database and updated PGDBs for the 480 *E. coli* strains described in this paper are all available at https://BioCyc.org. Access to EcoCyc is free; beyond a limited number of free monthly pageviews, access to other strains requires a paid subscription.

The ortholog-based annotation propagation software described in this paper is available as part of the Pathway Tools software, which is freely available for research purposes including source code, and available for a fee for commercial purposes. To obtain the software, see https://BioCyc.org/download.shtml.

The individual reports generated for many of the strains by the propagation software are included as Supplementary Material (file size limitations prevent inclusion of the full set of reports, but they are available upon request).

## Author Contributions

MK developed and ran the pipeline to compute ortholog pairs. SP developed the data-propagation software, performed the propagation, analyzed the results, and wrote the bulk of the manuscript. IK reviewed preliminary results and contributed suggestions to improve the methodology. PK provided guidance and oversight, and wrote part of the manuscript. All authors contributed to revising the manuscript, and approved the submitted version.

## Conflict of Interest

The authors declare that the research was conducted in the absence of any commercial or financial relationships that could be construed as a potential conflict of interest.
